# Investigation the effect of a herbal composition based on blackseed on patients with primary hypothyroidism: A randomized controlled trial

**DOI:** 10.22038/AJP.2024.23984

**Published:** 2024

**Authors:** Najmeh Javidi, Zahra mazloum khorasani, Roshanak Salari, Shabnam Niroumand, Mahdi Yousefi

**Affiliations:** 1 *Department of Persian Medicine, School of Persian and Complementary Medicine, Mashhad University of Medical Sciences, Mashhad, Iran*; 2 *Metabolic Syndrome Research Center, Mashhad University of Medical Sciences, Mashhad, Iran*; 3 *Department of Pharmaceutical Sciences in Persian Medicine, School of Persian and Complementary Medicine, Mashhad University of Medical Sciences, Mashhad, Iran*; 4 *Faculty of Medicine, Mashhad University of Medical Sciences, Mashhad, Iran*; 5 *Department of Persian Medicine, School of Persian and Complementary Medicine, Mashhad University of Medical Sciences, Mashhad, Iran*

**Keywords:** Primary hypothyroidism, Persian medicine, Traditional, Trachyspermum ammi L., Nigella sativa L., Citrus aurantifolia L.

## Abstract

**Objective:**

Hypothyroidism is characterized by insufficient production of thyroxine by the thyroid gland. Levothyroxine may not fully alleviate patients' symptoms. This study aimed to assess the impact of a herbal product on weight, body mass index (BMI), thyroid hormones, lipid profile, fasting blood sugar (FBS), depression, and quality-of-life scores in patients.

**Materials and Methods:**

72 patients with primary hypothyroidism, aged between 20 and 65 years old, participated in the trial and they were randomly allocated into two groups. The intervention group received the herbal powder containing *Trachyspermum ammi* L., *Nigella sativa* L., and *Citrus aurantifolia* L. while the control group received Avicel for 8 weeks.

**Results:**

Treatment with the herbal product resulted in statistically significant reductions in anthropometric variables such as BMI (p=0.03), hip circumference (HC) (p=0.008), waist circumference (WC) (p<0.001), and waist-to-hip circumference ratio (WHR) (p=0.003) in the intervention group in comparison between intervention and control groups. However, the decrease in weight was not statistically significant (p=0.08) in the intervention group compared the control group. In comparison between two groups, the depression score exhibited a statistically significant decrease in the intervention (p=0.001) and control groups (p=0.01), while there was a statistically significant increase in the quality-of-life score only in the intervention group (p<0.001) in comparison between intervention and control groups.

**Conclusion:**

The results indicate the potential beneficial effects of the herbal product on anthropometric variables in patients. Furthermore, the intervention yielded significant improvements in depression symptoms and quality-of-life scores among the patients.

## Introduction

Hypothyroidism is a prevalent disorder of thyroid hormone deﬁciency. Common symptoms of hypothyroidism include fatigue, lethargy, constipation, cold intolerance, weight gain, sound changes, and dry skin. Severe hypothyroidism without treatment can lead to death (Bai et al., 2018; Chaker et al., 2017). In the United States, 5 % of the population over 12 years old has hypothyroidism. The prevalence of disease in areas with sufficient iodine intake has been reported between 1-2% and up to 7% in adults. The prevalence of hypothyroidism in women is significantly higher than in men (Taylor et al., 2018; Tekieh et al., 2019). Primary hypothyroidism is the most common cause of hypothyroidism (Ke et al., 2015). Thyroid hormones affect most tissues in the body, controlling growth, maturity processes, and the action of some organs (Daza et al., 1997; Fisher et al., 1982; Roshangar et al., 2014; Wickenden et al., 1997). Hypothyroidism is a risk factor for heart disease and is associated with metabolic syndrome (Mehran et al., 2017). It affects the incidence and severity of depression and quality of life (Najafi et al., 2015) and is diagnosed biochemically with a high serum thyroid stimulating hormone (TSH) and low free T4. In most patients with hypothyroidism, TSH is more than 10 mIU/L (Chakera et al., 2012). Levothyroxine is the preferred treatment for hypothyroidism (Chakera et al., 2012; Tekieh et al., 2019). Despite the ease of use, high half-life, low cost, and acceptable complications, some symptoms are resistant to levothyroxine and it causes dissatisfaction in 10 - 15% of patients; T3 does not reach the normal range in 15% of patients (McAninch & Bianco, 2016). In 40% of levothyroxine users, TSH does not normalize (de Carvalho et al., 2018). Resistance to levothyroxine has been observed in some patients, which some researchers have emphasized on individual differences so it is necessary to pay attention to the role of Personalized Medicine based on the genotype in the treatment of uncontrolled patients (Jonklaas et al., 2014). Furthermore, long-term use of levothyroxine causes side effects (Dai et al., 1999; Monzani et al., 2001; Panebianco et al., 1997). According to the World Health Organization, 80% of people use traditional and complementary medicine for treatment (WHO, 2005; WHO, 2002). Treatment in Persian medicine is based on the temperament and nature of people. So, the use of plants with warm nature like *Trachyspermum ammi* L. and *Nigella sativa* L. may improve the symptoms of hypothyroidism.

 The effect of these plants has been evaluated on different diseases in human studies (Farhangi et al., 2016; Jalbani et al., 2016b; Tekieh et al., 2019; Ungjaroenwathana et al., 2019). The aim of the current study was to investigate the effect of combination of *Trachyspermum ammi *L.,* Nigella sativa *L. and *Citrus aurantifolia* L. on weight, laboratory tests, and depression and quality of life scores in hypothyroid patients with a BMI≥25 kg/m^2 ^(overweight and obese patients).

## Materials and Methods

**Study design**

This study was a Phase II, randomized, stwo armed, parallel, triple-blind, placebo controlled, superiority clinical trial. Sampling method was simple. Initially, participants signed the consent form and responded to the demographic information form, depression and quality of life questionnaires. Following this, biochemical, hormonal and anthropometric assays were conducted. Patients were divided into intervention and control groups using a blocked randomization model, with allocation concealment achieved through sealed envelopes. The randomization procedure was carried out completely blind by a researcher uninvolved in the clinical aspect of the study. The intervention duration was 8 weeks.

Out of the 122 patients evaluated for inclusion criteria, 80 were found to be eligible. Twenty patients did not meet the inclusion criteria, and 22 declined to participate. Furthermore, 5 participants were excluded from the intervention group due to itching, pregnancy, and dissatisfaction, while 3 participants were excluded from the control group due to stomach discomfort, pain, and dissatisfaction. Thus, a total of 72 patients completed the study.

Patients in the intervention and placebo groups received the herbal product powder or Avicel respectively for 8 weeks. The dosage of each plant and the study duration were determined based on previous studies (Farhangi et al., 2016; Jalbani et al., 2016 b; Ungjaroenwathana et al., 2019). Additionally, written lifestyle recommendations were provided to both groups in a standardized manner. The study commenced after receiving ethical approval from Mashhad University of Medical Sciences (IR.MUMS.REC.1400.037) and approval from Iran's Clinical Trials Registration Center (IRCT20210101049909N1), with adherence to the Declaration of Helsinki for the participation of human subjects in the study.

**Patients**

**Figure 1:**
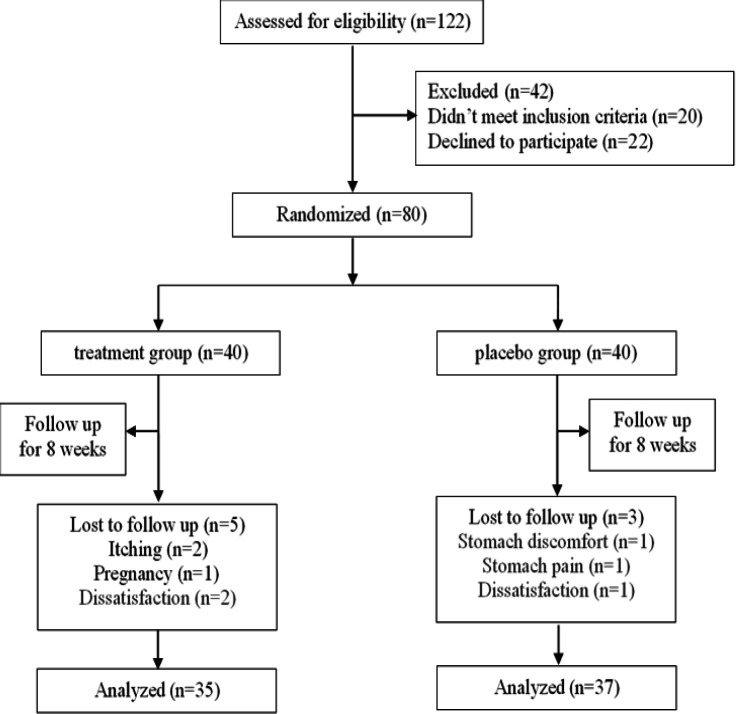
CONSORT Flow diagram of subject recruitment

In this controlled trial, 80 patients with primary hypothyroidism were enrolled (Figure 1). Subjects were recruited from the outpatient Endocrinology and Metabolism Clinic of Ghaem Hospital in Mashhad, Iran from July 2021 to September 2022. Inclusion criteria included individuals aged between 20 to 65 years, diagnosed with primary hypothyroidism by an endocrinologist, and classified as overweight or obese with a BMI≥25, whose weight had not been controlled with levothyroxine. Exclusion criteria consisted of uncontrolled systemic diseases, any recent weight loss diets, vitamin supplementation, patient dissatisfaction, pregnancy, and lactation.

**Drug and placebo preparation**

The plants and Avicel were purchased from reputable stores in Mashhad, Iran and authenticated by herbarium of Ferdowsi University of Mashhad with Voucher sp. No: E-1242 FUMH for *Trachyspermum ammi *L., Voucher sp. No: E-1241 FUMH for *Nigella sativa *L. and Voucher sp. No: E-1240 FUMH for *Citrus aurantifolia* L. After the plants were cleaned and the lemon kernels removed, a pure and soft powder was obtained through milling. Each patient in the intervention group received a daily 4.5 g herbal composition powder, consisting of 2750 mg *Trachyspermum ammi* L., 1000 mg *Nigella sativa* L. and 750 mg *Citrus aurantifolia* L. provided in 750 mg capsules. The control group received identical capsules containing Avicel. Participants were instructed to consume the herbal compound or placebo as 2 capsules three times daily after each meal. Furthermore, both groups used levothyroxine according to the endocrinologist's guidance.

**Variables**

Weight and BMI were the primary variables. Other measurements were free thyroxine (FT4), triiodothyronine (T3), thyroid stimulating hormone (TSH), fasting blood sugar (FBS), total cholesterol (CHL), high-density lipoprotein (HDL), low-density lipoprotein (LDL), triglyceride (TG), hip circumference (HC), waist circumference (WC), waist-to-hip circumference ratio (WHR), depression and quality of life. After measuring height, weight and blood pressure, patients provided blood samples at the Imam Reza Hospital Laboratory for the determination of the mentioned serologic indices. All variables were measured before and after the intervention. The serum and plasma samples were separated through centrifugation and stored immediately at −70°C. The reference values for TSH, free T4 and T3 were 0.4 to 6/1 µIU /ml, 10.3-25.8 pmol/L and 60 to 210 ng/dl, respectively. TSH, T3 and FT4 were measured by GAMA kit. Body weight and height were measured with a calibrated digital scale and measuring tape in centimeter, respectively. BMI by dividing weight (kg) to height (m) squared and WHR by dividing waist circumference (midway between the lowest rib and the iliac crest) (cm) to hip circumference (cm) were calculated. Finally, depression and quality of life were assessed using the Beck Depression Inventory-Second Edition (BDI-II) and the World Health Organization Quality of Life Questionnaire (WHOQOL-BREF) (Beck et al., 1996 ; WHOQOL, 2012). 

**Sample size calculation and statistical analysis**

The sample size was calculated based on two-sided chi-Square test and the following assumption: type Ì error=0.05, power of 80% and the risk difference equal to 0.3 according to the expert opinion. So, the sample size was calculated 35 participants in each group adding probable 10% drop out, 80 subjects entered to study. The data were analyzed by using SPSS 16 software. First, the characteristics of each group were described by descriptive statistics methods, including central and dispersion indices, according to the targets of the study and the investigated variables. Quantitative findings are shown as mean ± standard deviation (SD), and qualitative data are expressed as frequency and percent. Then, the chi-square test or Fisher's exact test was used to examine the qualitative variables between the two groups, and the t-test was used to compare the quantitative variables in the two groups if the data were normally distributed or the Mann-Whitney test was used if the data were not normally distributed. To compare the changes in quantitative variables such as weight, thyroid tests and biochemical tests before and after the intervention in each group, paired t-test or Wilcoxon test was used based on the distribution of data. In all statistics, p value<0.05 was considered statistically significant. 

## Results

A total of 122 patients were assessed for eligibility criteria, and out of these, 80 patients were found to be eligible for the study. Some subjects discontinued their participation due to various reasons including itching, pain and discomfort in the stomach, pregnancy, and dissatisfaction. Ultimately, 72 patients completed the study (Figure 1)

**General characteristics**

At the baseline, there were no statistically significant differences in the general characteristics between the groups (Table 1). 

**Primary outcomes**

Both the intervention and control groups demonstrated a reduction in BMI and weight. The reduction in BMI was found statistically significant in the intervention group (p=0.03), while in the control group, it was not statistically significant (p=0.16). The reduction in weight was not statistically significant in the intervention and placebo groups, respectively (p=0.084 and p=0.15 respectively) (Table 2).

**Table 1:** General characteristics of the subjects of the two groups

**Variable**	**Intervention** **N=35**		**Placebo** **N=37**		**p value**
Age (Years old)	42.69±9.943		40.70±8.518		0.366^*^
Gender	male	2(5.7%)	male	4(10.8%)	0.434^**^
	Female	33(94.3%)	Female	33(89.2%)	
Marital status	single	7(20%)	single	6(16.2%)	0.677^**^
	Married	28(80%)	Married	31(83.8%)	

Data are presented as mean±SD or number (percent)و^*^independent t-test.^**^chi-Square test

**Table 2:** BMI and weight of the subjects of the two groups before and after the intervention

**variable**	**Intervention** **N=35**	**Placebo** **N=37**	**p value**
Weight (kg)			
Before	81.64±14.58	79.76±12.45	0.55^*^
After	80.86±14.61	79.34±12.54	0.63^*^
p value	0.084^**^	0.15^**^	
BMI (kg/m2)			
Before	31.54±5.15	30.79±3.55	1^***^
After	31.24±5.24	30.63±3.63	0.63^***^
p value	**0.03** ^****^	0.16^**^	

BMI body mass index, Data are presented as mean±SD, ^*^independent t-test, ^**^paired t-test, ^***^Mann-Whitney test, ^****^Wilcoxon test, p value<0.05 is significant and bolded

**Secondary outcomes**

Significant reductions in waist circumference (WC) were observed in both the intervention and control groups (p<0.001). Hip circumference (HC) also significantly decreased in the intervention and control groups (p=0.008 and p=0.001, respectively). The waist-to-hip ratio (WHR) showed a statistically significant reduction in the intervention and control groups (p=0.003 and p=0.01, respectively) (Table 3). 

Fasting blood sugar (FBS) changes remained within the normal range in both the intervention and placebo groups. Total cholesterol demonstrated a non-significant decrease in the intervention group (p=0.84) while it increased in the control group. Low-density lipoprotein (LDL) showed a non-significant decrease in the intervention group (p=0.49) but increased in the control group. High-density lipoprotein (HDL) decreased in the intervention group while it increased non-significantly in the control group (p=0.13). Triglyceride (TG) levels increased in both groups. Thyroid-stimulating hormone (TSH) reduction was non-significant in the intervention (p=0.94) and control (p=0.33) groups. Additionally, free thyroxine (FT4) and triiodothyronine (T3) decreased in both groups (Table 4). 

**Table 3:** Anthropometric variables of the subjects of the two groups before and after the intervention

**variable**	**Intervention** **N=35**	**Placebo** **N=37**	**p value**
WC (cm)			
Before	99.83±11.27	98.08±9.34	0.47^*^
After	96.89±10.95	95.68±9.32	0.61^*^
p value	<0.001^**^	<0.001^**^	
HC (cm)			
Before	111.03±10.45	109.08±7.33	0.36^*^
After	109.74±10.96	107.89±7.16	0.81^***^
p value	0.008^****^	0.001 ^**^	
WHR			
Before	0.90±0.08	0.89±0.07	0.92^*^
After	0.88±0.08	0.88±0.07	0.91^*^
p value	0.003^**^	0.01^**^	

WC waist circumference, HC hip circumference, WHR waist to hip ratio. Data are presented as mean ± SD, ^*^independent t-test,^ **^paired t-test, ^*** ^Mann-Whitney test, ^***b*^ Wilcoxon test, p value<0.05 is significant and bolded

**Table 4:** Biochemical and hormonal tests of the subjects of the two groups before and after intervention

**N**	**Intervention** **N=35**	**Placebo** **N=37**	**p value**
FBS (mg/dl)			
Before	93.54±10.83	94.35±9.16	1^***^
After	95.90± 12.90	96.22±10.87	0.72^***^
p.value	0.05^****^	0.31^****^	
CHL (mg/dl)			
Before	190.20± 44.27	180.38±32.49	0.28^*^
After	189.03± 42.96	181.62±34.25	0.42^*^
p value	0.84^**^	0.75^**^	
LDL (mg/dl)			
Before	120.37±35.21	113.51±26.95	0.35^*^
After	117.21±36.24	114.76±26.39	0.75^*^
p value	0.49^**^	0.77^**^	
HDL (mg/dl)			
Before	42.60±10.73	40.30±9.03	0.32^*^
After	41.24±9.93	43.59±11.55	0.36^*^
p value	0.49^**^	0.13^**^	
TG (mg/dl)			
Before	141.86±100.67	127.51±50.49	0.23^***^
After	147.94± 97.98	135.78±69.90	0.54^*^
p value	0.35^****^	0.25^**^	
TSH (µIU /ml)			
Before	4.27±3.44	4.26±6.53	1^***^
After	4.20±5.04	3.47±3.36	1^***^
p value	0.94^**^	0.33^****^	
FT4 (pmol/l)			
Before	16.65±5.31	16.58±5.84	0.96^*^
After	14.69±5.49	16.18±5.92	0.28^*^
p value	0.07^**^	0.90^**^	
T3 (ng/dl)			
Before	158.22±30.99	144.18±27.16	0.14^***^
After	147.52±33.64	140±36.30	0.37^*^
p value	0.10^****^	0.59^**^	

^*^Independent t-test, ^**^paired t-test, ^***^ Mann-Whitney test, ^**** ^Wilcoxon test. Data are presented as mean±SD, p value<0.05 is significant

The depression score significantly decreased in both the intervention and placebo groups (p=0.001 and p=0.01, respectively), On the other hand, the quality-of-life score exhibited a statistically significant increase in the intervention group (p<0.001) and a non-significant increase in the control group (p=0.54) (Table 5).

Considering the higher prevalence of hypothyroidism in women, we conducted a re-analysis by excluding data from male participants. Since the majority of the participants were women, this exclusion did not affect the results (Table 6).

The results indicate that there was no statistically significant difference between the two groups in any of the variables.

**Table 5:** Quality-of-life and depression scores of the subjects of the two groups before and after the intervention

**N**	**Intervention** **N=35**	**Placebo** **N=37**	**p value**
quality-of-life score			
Before	62.85± 15.60	65.52± 18.28	0.95^***^
After	70± 16.09	67.22± 18.24	0.92^***^
p value	**<0.001** ^****^	0.54^****^	
depression score			
Before	14.37± 11.24	14.30± 11.10	0.99^***^
After	11.09± 9.37	10.51± 9.57	0.79^*^
p value	**0.001** ^****^	**0.01** ^****^	

^*^Independent t-test, ^**^paired t-test, ^***^ Mann-Whitney test, ^****^ Wilcoxon test. Data are presented as mean±SD, p value<0.05 is significant and bolded

**Table 6:** Weight, BMI, WC, HC, WHR of the subjects of the two groups before and after the intervention in female participants

**N**	**Intervention** **N=33**	**Placebo** **N=33**	**p value**
Weight (kg)			
Before	80.45±13.02	77.84±9.63	0.35^*^
After	79.70±13.32	77.53±9.84	0.45^*^
p value	0.08^**^	0.30^**^	
BMI (kg/m2)			
Before	31.54±5.21	30.76±3.43	0.47^*^
After	31.25±5.33	30.63±3.51	0.58^*^
p value	0.08^**^	0.29^**^	
WC (cm)			
Before	99.48±10.94	97.09±8.76	0.33^*^
After	96.55±10.82	94.73±8.74	0.45^*^
p value	**0.000** ^**^	**0.001** ^**^	
HC (cm)			
Before	111.21±10.51	108.82±6.85	0.27^*^
After	109.94±11.11	107.85±6.82	0.36^*^
p value	**0.006** ^**^	**0.002** ^**^	
WHR			
Before	0.89±0.08	0.89±0.07	0.83^*^
After	0.88±0.08	0.87±0.07	0.92^*^
p value	**0.003** ^**^	**0.01** ^**^	

^*^Independent t-test, ^**^paired t-test, Data are presented as mean±SD. p value<0.05 is significant and bolded

## Discussion

In this study, the intervention group showed a statistically significant decrease in anthropometric variables, including BMI, HC, WC, and WHR. However, there was no statistically significant decrease in weight. Additionally, meaningful changes in depression and quality of life scores were observed in the intervention group, while biochemical (total cholesterol and LDL) and hormonal (TSH) parameters showed nonsignificant reductions.

 The composition used in this study was not similar to other studies, so the results of this study cannot be compared exactly with other results. But there were studies that contained some components of the composition, some of which are mentioned. for example in an animal study using only blackseed conducted by Khalawi et al. (2013), the reduction of TSH was similar to our study and the reduction of thyroxine is in accordance to the study of Al-Asoom et al. (2014) in male Wistar rats. Some findings are in agreement with similar human studies. Reduction of TSH and anthropometric factors were also reported in Farhangi et al.'s study (2016) with *Nigella sativa* L. in 40 Hashimoto’s thyroiditis patients. In Farhangi et al.’s study, weight loss was significant and reduction of WHR was not significant in the intervention group while in our study, weight loss was not significant but WHR reduction was significant. Also, in Fatemi et al.'s research (2019) with black seed in 42 patients of all types of hypothyroidism CHL and FBS reduction was significant in drug group with negative anti-thyroid peroxidase (ATPO) antibody, while in our research reduction of CHL was not significant and FBS changes were within the normal range in the intervention group. Therefore, we conducted the first human study with this herbal product in 72 primary hypothyroid patients.

*Trachyspermum ammi* L. also known as carum copticum or ajwain, belongs to the Apiaceae family. Ajwain grows in many areas in the world and Iran (especially Baluchistan). It has been used in traditional Persian medicine for many years. This plant has warm nature and different therapeutic effects include broncho-dilatory, antitussive, anti-dyspnea, carminative, antiseptic, amoebiasis expectorant, antimicrobial, antiparasitic, antiplatelet-aggregatory and antilithiatic. Also, it is effective in treating common cold and acute pharyngitis (Boskabady et al., 2014; Zahin et al., 2010; Zarshenas et al., 2013).

*Nigella sativa *L*. *or black seed from the Ranunculaceae family is an annual herb with extensive range of therapeutic properties including antioxidant, anti-inflammatory, anti-diabetic, anti-cancer, analgesic, anti-microbial, spasmolytic, bronchodilator, hepato-protective, renal protective, gastro-protective and immune-modulatory effects and no side effects were reported used doses . Also, it is useful in treatment of some diseases such as diabetes, hyperlipidemia, hypertension and gastritis (Tekieh et al., 2019; Ahmad et al., 2013; Farhangi et al., 2016; Islam et al., 2017; Shariatifar et al., 2014).

*Citrus aurantifolia *L*.* belongs to the Rutaceae family. It is usually known as key lime or bitter orang. Lime is widely used in West Africa, especially in Nigeria. It has many uses in herbal medicine. It is an essential substance in the most herbal concoction. Studies have shown bioactive activities for this plant in cold fevers, sore throats, sinusitis, bronchitis and asthma. Lime oil is helpful for depression, rheumatoid arthritis, obesity, acne, herpes, cuts and insect bites. It is also used as a cleansing for oily skin and acne (Aibinu et al., 2006; Khan Pathan et al., 2012).

Today, medicinal plants play a significant role in curing and controlling diseases and they have been able to help a lot in reducing symptoms and improving the condition of patients. The existence of scientific evidence is proof of this claim (Ardakani Movaghati et al., 2019; Derakhshan et al., 2019; Ghazanfari et al., 2022; Hamidnia et al., 2018; Yousefi et al., 2016).

 Temperament refers to the quality resulting from the interaction between opposing qualities.

According to Persian traditional medicine, health is characterized by the normal functioning of all activities, free from any disorders or defects. When the temperament is imbalanced, illness may arise (Avicenna, 2005). Symptoms of a cold-dominant temperament include cold sensation, excess fat, reduction in lean body mass, pallor, slow psychomotor, timidity, little appetite, weak and slow digestion, low sexual desire, excessive sleep, weak pulse. Also, premature aging and drowsiness in the face are the characteristics of cold dominance. Symptoms of a wet-dominant temperament include soft skin, lax muscles, decreased nerve conduction velocity, low energy and fatigue sensation (Naseri, 2009; Avicenna, 2005; Chashti, 2008; Ahwazi, 2008). The combination of cold and wet symptoms is called cold and wet dominance. From the perspective of Persian medicine, hypothyroidism is compatible with increasing coldness and humidity in the body. The viscosity of body fluids increases in hypothyroidism, so plants such as carum copticum and black seed can adjust the density and consistency of body fluids and make the body temperament to balance (Naseri, 2009). Therefore, in design of this herbal product, in addition to black seed, ajwain and *Citrus aurantifolia *L. were also used in order to benefit from their effects in treatment of hypothyroid patients. These medicinal plants have synergetic effects and cover the pathways of pathogenesis. 

According to studies, black seed and its active component thymoquinone have potential mechanisms for weight loss, include reducing lipogenesis, increasing lipolysis, and enhancing satiety and fullness (Hasani-Ranjbar et al., 2013; Qidwai et al., 2009). Research has shown that *Trachyspermum ammi* can lead to a reduction in BMI and improvement in lipid profile. This may be attributed to the effects of thymol, the main ingredient in Trachyspermum, which can lower the activity of the liver enzyme 3-hydroxy-3-methylglutaryl coenzyme A (HMG-CoA) reductase involved in cholesterol synthesis or inhibit lipase, thereby reducing cholesterol absorption from the gastrointestinal tract (Yaqoob et al., 2022).

 The findings show significant weight loss in obese mice by administration of *Citrus aurantifolia*. Some components like polyphenol and flavonoids inhibit lipogenesis or induce lipolysis. Accumulation of lipid decrease afterward decrease adipocytes. there is little evidence that *Citrus aurantifolia* can directly affect adipogenesis in white adipocytes and thermogenesis in brown adipocytes which are both important targets of anti-obese strategy (Park et al., 2019).

In some studies, the positive effect of *Trachyspermum ammi* L.,* Nigella sativa *L. and *Citrus aurantifolia *L. in reducing weight, BMI and anthropometric characteristics has been observed (Hashemipour et al., 2016; Mahdavi et al., 2015; Yaqoob et al., 2022). 

Also, studies show that the use of black cumin and key lime is effective in reducing depression (Zadeh et al., 2022; Zare et al., 2019). Improving the quality of sleep with *Citrus aurantium* and increasing the quality of life with black seed and ajwain in some diseases are other scientific achievements (Abbaspoor et al., 2022; Alizadeh‐naini et al., 2020; Hatami et al., 2020). Yazdian et al. in their study found that carum copticum seeds have a soothing effect on gaseous symptoms in patients with bloating and flatulence (Yazdian et al., 2014). Also, improvement in LDL and HDL cholesterol level with ajwain in primary hyperlipidemic patients was reported (Jalbani et al., 2016a). The presence of biologically active constituents in *Citrus aurantifolia* L. has caused its wide role in treatment of diseases (Khan Pathan et al., 2012; Suryawanshi, 2011). In Persian traditional medicine, hypothyroidism is believed to be caused by the accumulation of cold and wet substances in the body, leading to a decrease in basal metabolic rate and thyroid function. Digestive problems and indigestion contribute to the accumulation of these substances. Therefore, plants with a warm temperament can help balance these substances, increasing basal metabolism and improving thyroid function. Carum copticum and black seed, being warm in temperament and they can alleviate digestive problems in hypothyroid patients. *Citrus aurantifolia* L. helps to regulate blood and other body fluids' viscosity and acts as a temperament regulator in the presented herbal composition (Aghili, 2009; Avicenna, 2010; Chashti, 2008).

 The diagnosis of hypothyroidism is based on laboratory findings and the treatment is done by hormone replacement. Set point of thyroid homeostasis determined by the various genetic, epigenetic and allostatic (obesity, age, pregnancy, and non-thyroidal illness) parameters. The relationships between TSH and thyroid hormones are individual, dynamic, and adaptive that all these factors are effective in linking thyroid stimulating hormone and thyroid hormone regulation and affect the pattern of diagnosis and treatment of hypothyroidism (Hoermann et al., 2017). Also dietary factors (Mezzomo and Nadal, 2016; Pałkowska-Goździk et al., 2018) and massage (Field, 2016) interfere with the treatment of disease. Persian medicine emphasizes lifestyle modifications as the first step in treatment, including climate adjustment, nutrition, exercise, sleep pattern, waste elimination, and stress management tailored to each patient's temperament (Ansaripour et al., 2019; Sina, 2005).

Based on the principles of Persian medicine, future studies are suggested to provide precise lifestyle instructions considering the patients' temperaments, calculate drug dosage based on weight, and extend the duration of the studies.

The limitations of this study include factors such as diet, activity level, a wide range of patient weights, the high prevalence of depression in patients, fixed drug dosage, and the duration of the intervention.

Herbal combination (*Trachyspermum ammi *L.*, Nigella sativa *L.*, Citrus aurantifolia *L.) had a significant effect on anthropometric factors, depression, and quality of life scores in patients with primary hypothyroidism and BMI≥25. However, the effect size observed was very small. Nonetheless, our intervention could be considered as an effective adjuvant treatment for hypothyroid patients. It is recommended that future research focus on studies with attention to lifestyle modifications and longer intervention duration.
